# 

**Published:** 1910-12-31

**Authors:** 


					Just Issued. Ninth Edition, Rewritten and Enlarged, 9s. net.
PHARMACY, MATERIA MEDIGA & THERAPEUTICS.
Being a Commentary on tlie B.P. and its Drugs, with their Preparations,
and on all the new Non-official Remedies in use, with Chapters on the
different processes used in Dispensing, Prescription-writing, Latin
Glossary, &c. By Sir W. WHITLA, M.D., LL.D., Professor, Materia
Medica, Queen's University, Belfast, and Senior Physician, Royal Victoria
Hospital.
By the same Author. New Work. 1,900 pages. Crown 8vo. 25s. net.
PRACTICE OF MEDICINE.
Containing in two Handy Volumes a complete and independent Article on
the Etiology, Symptoms, and Diagnosis of every Medical Disease; arranged
in Dictionary Form for Practitioners and Students.
London: BailliiSre, Tindall & Cox, 8 Henrietta Str??t, T?.0.
Crown 8vo. paper cover, 6d.
ON PREPARATION FOR OPERATION IN
PRIVATE HOUSES.
By STANMORE BISHOP, F.R.C.S.
THE SCIENTIFIC PRESS, LTD.,
28 & 29 SOUTHAMPTON STREET, STRAND, LONDON, W.O.
The FOTIfRE of tie HOSPITALS.
Medical Relief for All according to means.
A Scheme of Co-operation between the PATIENTS, the
HOSPITALS, and the STATE.
By Sir HENRY BURDETT, K.C.B., K.C.V.O.
Price 2d.
THE SCIENTIFIC PRESS, LTD.,
28 & 29 Southampton Street, Strand, London, W.C.
And at all Bookstalls.
TWO USEFUL HANDBOOKS FOR
SECRETARIES.
HOSPITAL EXPENDITURE: THE COMMIS=
SARIAT. For Hospital Matrons and Managers.
2/6, post free.
" This should prove a valuable handbook to those upon whom rests
the responsibility of hospital finance, and should be of interest to the
public as giving a fair idea of what this department should and does
cost."?Manchester Courier.
A LEGAL HANDBOOK FOR THE USE OF
HOSPITAL AUTHORITIES. By Leonard
Syer Beistowe, M.A. Oxon., Barrister-at-Law.
2/6, post free.
THE SCIENTIFIC PRESS, Ltd.
28/29 SOUTHAMPTON STREET, STRAND, LONDON, W.C.
Graves' Disease. ^
In cases where there is a large goitre in Graves''
disease I usually employ some preparation of iodine.
Iodide of potassium is useful, but I more often em-
ploy the syrup of hyriodic acid or iodipin. This
latter compound may be used both internally and
externally.?Dr. Hector Mackenzie.
Diet and Thoracic Disease.
There is no question in my mind that almost ali
the bronchitis and much of the broncho-pneumonia,
in babies are entirely due to bad feeding, and it has
been pointed out that in the Midlands, where the-
working people live .largely on bread and butter and'
tea, a great deal of the chronic bronchitis from which
they suffer is due to their defective diet. And its
occurrence may also, in a large measure, be pre-
vented by suitably altering their diet. The influence
of diet in the production of asthma is also a matter
of importance. From post-mortem examinations it
not infrequently appears that great harm has been
done by misdirected energy in feeding, the stomach
being sometimes enormously distended with large
quantities of egg and milk, given in cases of pneu-
monia or in severe heart disease.?Dr. G. Newton
Piit.
Appointments and Resignations.
Mr. R. Godwin Chase, M.B., B.C.Cantab., etc., has
been appointed honorary surgeon to the Chesterfield and
North-East Derbyshire Hospital.
Dr. James Mackenzie, M.D. Edin., Hon. LL.D. Aber.,
has been appointed Lecturer on Cardiac Research at the
London Hospital Medical School.
Mr. A. H. Burgess, M.B., M.Sc., F.R.C.S., at present
Lecturer in Systematic Surgery at Manchester University,
has been appointed to the Lectureship in Practical Surgery
vacant through the death of Mr. J. E. Piatt.
Messrs. Rowntree and Co., Ltd., of York, have been
appointed makers of cocoa and chocolate to his Majesty
King George Y. and also to her Majesty Queen Mary.
This is the third time that sovereigns of the British Empire
have honoured the manufacturers of "Elect" cocoa with
Royal Warrants. Rowntree's cocoa is well known, and
deservedly appreciated in all hospitals.
BOOKS RECEIVED.
Kegan Paul, Trench and Co.
" Medicine and, the Church." By J. Rhodes.
" Legend of Our Lord." By Mrs. Arthur Bell.
" Medical Guide." By H. Morre.
" The Saviour of the World." By Miss C. M. Mason.
Allenson.
" The Faith of the Evolutionist." By Palm.
T. Fisher Unwin.
" Woman and Marriage." By Margaret Stephens.
" Aspects of Death in Art." By F. P. Weber.
In acknowledging the receipt of a further grant of ?2,500
from, the Salford King Edward Memorial Fund, Mr.
Frederick Burton, the Treasurer of the Salford Royal Hos-
pital, says the hospital authorities are delighted to learn
that over ?7,000 has now been contributed to the Memorial
Fund.
422 THE HOSPITAL December 31, 1910.
Gifts and Bequests.
The Queen has sent ?10 and a gift of pheasants to
the British Home and Hospital for Incurables at Streat-
ham.
Mrs. Catherine Schmidt, of Fulford, near York, who
left estate valued at ?50,323 gross, has bequeathed ?1,000
to the Cameron Hospital, West Hartlepool, in augumenta-
fcion of the endowment iund and not to be used as income.
Her Majesty the Queen and her children have sent a
gift of toys to the younger patients and a number of
pheasants to the adults at the Royal Hospital, Richmond ;
and a gift of pheasants has also been received at St. George's
Hospital.
Lady Juliet Duff has received the following further con-
tributions towards the fund for paying Charing Cross
Hospital mortgage : Lady Wernher, ?100; the Rev. F. A.
Minnitt, ?21; Mr. S. Heilbut, ?10 10s. ; Mr. James Watt,
?5 5s. ; Mrs. R. A. B. Preston, ?5 5s.; Lady Edith Cotes,
?5; and Mrs. George Turner, ?5.
Mrs. Sophia Merrall, of Law House, Haworth, Yorks,
who left estate of the gross value of ?105,505 lis. 10d.,
has left her ordinary shares in Merrall and Son (Limited)
ultimately, in certain contingencies, to local charities, in the
discretion of her executors, requesting them to bear in
mind the endowment of certain churches and of the
Keighley Victoria Hospital.
Mr. Edward Henry Parker, professionally known as
Lester Collingwood, of the Alexandra Theatre, Birming-
ham, theatre proprietor and touring manager, who left
estate of the gross value of ?12,155 Is. Id., has left ?100
each to the Women's Hospital, Birmingham; the Women's
Hospital, Sparkhill, Birmingham; the Birmingham General
Hospital; the Queen's Hospital, Birmingham; and to the
Children's Hospital, Birmingham.
According to the will of the late Mr. Joseph Henry
Houldsworth, ironmaster, of Glasgow, 10 per cent, of the
residue of his estate is to be divided among charitable
institutions, at the discretion of his trustees, except eo far
as the testator might himself have made the allocation.
This allocation, made in a codicil up to ?20,000, includes :
?5,000 to the London Homoeopathic Hospital, Great
Ormond Street; ?1,000 each to the Glasgow Royal Infir-
mary ; the Western Infirmary, Glasgow; the Victoria In-
firmary, Glasgow; the Broomhill Home for Incurables;
the Royal Hospital for Sick Children, Glasgow; the Ayr
County Hospital; and the Royal Scottish Hospital in
London.
Miss Marian Julia James, of Hindhead, Surrey, who
left estate of the gross value of ?92,240 2s. 2d., has be-
queathed ?500 to St. Thomas's Home in connection with
St. Thomas's Hospital; ?200 to the Hospital for the Study
and Treatment of Obscure Diseases at Hartington Grove
Hills Road, Cambridge; ?200 to Aneurin Williams
in trust to help to found a museum at the First Garden
City at Letchworth. Subject to other bequests and pro-
visions the testatrix left the residue of her estate upon
trust as her residuary legatees shall determine. Miss
James's object was shortly to provide and maintain hostels
or boarding-houses where, primarily, educated persons of
the professional or business classes and of limited means
in need of a holiday or rest may obtain accommodation at
inexpensive charges and according to reasonable standards
of refinement and comfort, and to provide hospitals for
persons of .the classes indicated, and in other ways to
minister to their comfort.
The Rev. Hugh Pearson, of Cheltenham, has left ?100
to the Home for Sick Children, Battledown, Cheltenham.
Mr. Charles Gaunt, of Farsley, Yorks, woollen and
worsted manufacturer, has bequeathed ?100 to the Leeds
General Infirmary.
Miss Elizabeth Perks, of St. George's Square, Wor-
cester, has bequeathed ?1,000 to the Worcester General
Infirmary and ?500 to the Worcester City and County
Nursing Institute.
The Worehipful Company of Skinners has given ?10
to the After Care Association for poor persons discharged
recovered from asylums for the Insane, Church House,
Dean's Yard, Westminster, S.W.
Mr. Henry William Trinder, of Bishops Waltham,
Hants, has left ?300 to the East London Hospital for
Children, Shadwell; ?100 each to King's College Hospital
and to the Royal Hants County Hospital, Winchester.
Mrs. Wynyard, of Clarendon Road, Holland Park,
W., widow of Mr. Clinton Wynyard, sometime British
Consul at Riga, left estate in the United Kingdom of the
gross value of ?51,344. She bequeathed ?200 to the
Building Fund of St. Bartholomew's Hospital.
Mr. James Clifford, of Oxford, retired stone and
memorial mason, has bequeathed ?1,000 each to the Rad-
cliffe Infirmary for the endowment of a bed to bear his
name, and the Oxford Eye Hospital, on condition that the
bequest shall bear his name.
Mr. John Stafford, J.P., and Mr. E. T. Hutchinson,
of Leicester, have each bequeathed a legacy of ?100 to
the Leicester Infirmary, which also is the residuary legatee
of Mrs. Emma Dyke Frost, of Leicester, whose residuary
estate ie estimated at about ?3,000.
Lord Sandhurst, treasurer of St. Bartholomew's Hos-
pital, has received from Sir Henry Harben, in memory of
the late Mr. Henry Harben, ?500 for the general funds
of the hospital and ?500 for the Nurses' Home Rebuilding
Fund, and from Mr. Richard Foster ?105 towards the
special fund for paying the existing debt.
The Lord Mayor handed to the chairman of the Middle-
sex Hospital, Prince Alexander of Teck, a cheque
for a hundred guineas from the City Corporation to the
Prince Francis of Teck Memorial Fund for the endowment
of the hospital. Mr. Walter Emden intends to continue
his annual gift of ?100 to maintain the scholarship attached
to the Cancer Research Department in connection with the
Cancer Charity of the hospital.
Prince Alexander of Teck has received the following
additional contributions to the Prince Francis of Teck
Memorial Fund for the endowment of the Middlesex Hos-
pital : Mrs. C. E. Rube, ?52 10s.; Lieut.-Colonel R. H.
Carr-Ellison, ?20; Mr. and Mrs. Moreing, ?25; the Earl
and Countess of Scarborough, ?10 10s. ; and " Anony-
mous," ?10; Lord Wemyss, ?25; Lady Paget, ?20;
" Anonymous," ?25; the Fishmongers' Company, ?105; the
Gold-smiths' Company, ?50; Mr. Bradley Martin, ?52 10s.;
and " R. M.," ?40.
Mr. Frederick Bloomenthal, of Pimlico, wholesale
stationer, of Cannon Street, has bequeathed the determina-
tion of three life-interests, the ultimate residue of his
property, as to one-sixteenth to the following among other
charities : The London Hospital, Guy's Hospital, St.
Bartholomew's Hospital, St. George's Hospital, the West-
minster Hospital, the Charing Cross Hospital, the Gray's
Inn (? Royal Free) Hospital, the Cancer Hospital, the
Brompton Hospital for Consumption, and St. Thomas's
Hospital. It would appear that the amount eventually
available for charitable purposes will amount to nearly
?30,000.
December 31, 1910. THE HOSPITAL 423
The Queen has sent ?10 to the Hospital for Women,
Soho Square.
The Girdlers' Company has distributed ?400 among
certain hospitals and charitable institutions in London.
Mrs. Lydia Ciiowther has bequeathed ?200 each to
the Stourbridge Dispensary and the Kidderminster
Infirmary.
Miss Mariam Tomlin, of Langley House, Prestwich,
Lanes, who died on December 11, aged eighty-one, left
?100 to the Nottingham Dispensary.
The Queen has sent gifts of pheasants to the National
Hospital for the Paralysed and Epileptic (Albany Memorial),
Queen Square, Bloomsbury, the Friedenheim Hospital,
Swiss Cottage, the British Lying-in Hospital, Endell Street,
and Queen Charlotte'*; Hospital.
Miss Isabella Louisa Boustred, of Blackheath, has
bequeathed, subject to the interest of her sister, ?1,000
to the Seamen's Hospital Society, Greenwich; ?2,000 to
St. John's Hospital, Blackheath; ?200 each to the Royal
Hospital for Incurables and the Hospital for Sick Children,
Great Ormond Street; and ?100 to the Deptford Medical
Mission. The residue of her estate, subject to some specific
bequests, she left to Guy's Hospital.
St. Mary's Hospital, Paddington, has received a fur-
ther donation from Sir Henry Harben of ?1,000, in
memory of the late Mr. H. A. Harben, and also dona-
tions of ?20 from the Saddlers' Company and ?10 from the
Football Association.
The Fishmongers' Company has granted ?105 each to
the Prince Francis of Teck Memorial Fund (Middlesex
Hospital), and Homes for Little Boys, Farningham; to
the London Lying-in Hospital, ?52 10s. ; to the London
Hospital, ?52 10s.; to the Royal Free Hospital, ?21; to
the Southwark, Newington, and Walworth Nursing Asso-
ciation (Fishmongers' Company's Nurse), ?105; and to the
Brompton Hospital for Consumption, ?31 10s.
Sir George Sutherland Mackenzie, of Cadogan Square,
S.W., and of Tempsford Hall, Bedford, formerly adminis-
trator of the Imperial British East Africa Company's
Territories, who left estate of the gross value of
?104,004 19s. 6d., has bequeathed the residue of his pro-
perty to his children in equal shares, and should he leave
no children to the Ross and Cromarty County Committee
?2,000 on trust to found two bursaries each of the value of
?30 per annum, to be known as the " Sir William Mackenzie
and the Jessie Mackenzie Inchvannie Bursaries." (after his
father and his aunt), to be tenable at the Scottish Univer-
sities for students from Ross and Cromarty, and prefer-
ably from the National Schools, for the study of medicine ;
to his brothers James and William ?30,000 for life, with
remainder to the president and council of the Royal College
of Physicians and the Royal College of Surgeons, England,
for the endowment of scientific research by students of
ability and of registrable medical qualification, who may
thus be able to devote their whole energies to such work,
and be independent of ordinary practice. He made this
bequest in the hope that the combined results of the
systematic work of so many trained workers may prepare
the way for a genius to come who will make great dis-
coveries, and he also stated that this bequest was in memory
of Sir William MacKinnon, baronet, to whose kindness and
influence in earlier years he owed both the means and the
inclination to make the gift.
Miss Amelia Busii, of Clifton, has bequeathed ?200 to
the West of England Sanatorium at Weston-super-Mare.
Mr. John Lovett, of Lewie Road, Neath, licensed vic-
tualler, has bequeathed ?50 to the Swansea General and Eye
Hospital.
Miss Frances Harrison* of Leven, Yorks, has be-
queathed ?500 eaeh to the St. James's Convalescent Home,
Bridlington, and to the Bridlington Cottage Hospital.
Mrs. Edith Annie Freeman, of Bilton Court, Knares-
borough, who left estate valued at ?110,230 gross, has-
bequeathed ?1,000 to the Halifax Infirmary and ?500 to
the Royal Bath Hospital and Convalescent Home, Harro-
gate.
The Marquis of Bute has promised to subscribe 5,000
guineas to the fund for the Cardiff City memorial to King
Edward VII. This is perhaps the largest response which
has been received to the appeal of the board of manage-
ment of Cardiff Infirmary. The directors of the Lewis
Merthyr Collieries have also contributed 1,000 guineas.
Mr. Henry Lucas, of Gloucester Square, Hyde Park, and
East Grinstead, barrister-at-law, has bequeathed ?500 to
University College Hospital, and ?100 to the East Grin-
stead Cottage Hospital. He expressed the desire that
these bequests for charitable purposes should be invested
by the recipients, and the income only applied.
Sir William Treloar has received from Prince Henry
the sum of ?1 3s. and from Prince George ?1 Is., their
annual collection, which they have sent as members of
the Queen Alexandra League, an association of children
banded together to assist the Cripples' Home and College
at Alton. The League has over 5,000 members, and is not
confined to boys and girls in the United Kingdom.
Mrs. Isabella Foster, of Woodcote, Newport, Salop,
bequeathed ?100 each to the Universal Beneficent Associa-
tion, the Infant Orphan Asylum at Wanstead, the Royal
Albert Orphanage, the Royal Hospital for Incurables,
Putney, the Governesses' Benevolent Institution, and the
National Benevolent Society; and ?50 each to the Men's
Convalescent Society and the Women's Convalescent Home
at Rhyl.
Mr. John Bradshaw, of Cheltenham, who left estate
valued at ?92,795 gross, has bequeathed ?1,000 to the
Lancaster Royal Infirmary to endow a "John Bradshaw "
bed; ?500 to the Royal Infirmary, Oxford Road,
Manchester; ?300 to the Home for Incurables, Harro-
gate; ?200to the Cheltenham General Hospital; and ?100
to the Harrogate Infirmary (formerly the Cottage Hos-
pital).
According to this week's Nursing Mirror the
members of the nursing staff at Cumberland Infirmary,
Carlisle, were busily engaged in the decorations for
Christmas, when a telephone message came through stat-
ing that a serious railway accident had occurred down
the line, and asking the authorities to prepare for the
admission of a number of patients. No details were
given. There were a few beds vacant in the men's wards,
but none in the women's. However, by utilising couches,
which often ha3 to be done at Cumberland Infirmary, it
was hoped that adequate accommodation would be pro-
vided. This was done in less than a quarter of an hour,
but unhappily only one of the beds was needed, the rest
of the victims of the disaster having passed away before
medical aid could be rendered.
424 THE HOSPITAL December 31, 1910.
Jottings.
Queen Amelia of Portugal, attended by Pr. Recamier,
?visited the Evesham Cottage Hospital this \v . ek, and, after
inspecting the wards, congratulated the latron on the
improvements which have been made since her former
visit.
A new hospital motor-boat, named the Yale, and built
by private subscription under the auspices of the "Yale
Grenfell Association " for use of the Mission in Labrador,
lias been duly delivered in good condition and free from
debt, with the insurance paid for one year in advance.
During the past year the ambulance service of the
Metropolitan Asylums Board removed 14,474 patients
suffering from fever, diphtheria, or smallpox from their
homes to the Board's London hospitals, 6,014 from those
hospitals to convalescent hospitals in the country, and
6,068 from the convalescent hospitals to London on
recovery. ,
The Marchioness of Londonderry (Mayoress of Durham)
visited the Durham County Hospital and the workhouse
recently, and held an "At Home" in the Town Hall
afterwards. Among the guests were Mrs. Kitchin [wife
of the Dean of Durham), Mr. J. W. Hills, M.P., Captain
Apperley, the Deputy-Mayor (Councillor W. H. Wood),
Mr. and Mrs. Ralph Simey, and Dr. and Mrs. Gee.
At Hackney on Saturday last an inquest was held on a
man, aged seventy-nine, who had recently attended as an
out-patient at the London Hospital for an internal growth.
X)n Wednesday, December 14, he went into the Hackney
Infirmary, and had been placed in a room without a
fire and in a shirt with only one button on it. A
former infirmary sick nurse said the deceased was taken
into the mental ward, where he had been knocked down
in the bed and shaken and struck across the face with a
pillow. This evidence was contradicted by other wit-
nesses. The Coroner said that the Guardians had sent a
communication to the effect that the matter would receive
the most careful attention of the board. The jury found
that the deceased died from natural causes, but expressed
the opinion that the allegations made against the infirmary
.officials should be fully investigated.
Tiie Corporation of Glasgow has accepted the following
tenders for work in connection with (1) an extension of
the Avenue Home at Belvidere Hospital; (2) additions to
the administrative block at Belvidere Hospital; and (3)
alterations on the day workers' block at Ruchill Hospital :
^1) Mason work, Messrs. Brown, Fraser and Co., 59 Blue-
vale Street, Glasgow (?758 15s. lOd.) ; joiner work,
Messrs. Duncan Buchanan, 399 London Road, Glasgow
(?642 19s. lid.); tile work, Mesrs. Haddow, Forbes and
Co., Ltd., 140 Bath Street, Glasgow (?114 18s.) ; plaster
work, Messrs. Graydon and Miller, 29 Craignestock Street,
Glasgow (?121 Is. 8d.); heating work, Messrs. James
Combe and Son, 103 North Hanover Street, Glasgow
(?199 3s. lid.). (2) Mason work, Messrs. Brown, Fraser
and Co. (?2,506 9s. 2d.); joiner work, Messrs. Duncan
Buchanan (?1,240 6s. 2d.); plaster work, Messrs. Graydon
and Miller (?252 7s. 8d.); heating work, Messrs. James
Combe and Son (?198 15s. 2d.); tile work, Messrs. Had-
-dow, Forbes and Co. (?141 3s. Id.); slater work, Messrs.
J. M'Quat and Son (?108). (3) Mason work, Messrs.
Brown, Fraser and Co. (?537 Is.) ; joiner work, Messrs.
Duncan Buchanan (?670 6s. 5d.); plumber work, Messrs.
R. Munro and Son, 81 North Keppochhill Road, Glasgow
<(?380 17s. 6d.); plaster and tile work, Messrs. Haddow,
Forbes and Co. (?381 4s. 3d.).
The Queen has sent five brace of pheasants to the
London Lock Hospital and Rescue Home, Harrow Road,
Paddington, W.
The concert given at the Midland Hotel, Manchester,
in aid of the Ancoats Hospital Appeal Fund, arranged by
Miss Nesbitt, realised ?29.
Miss Alys Bateman is arranging a performance of
Verdi's "Requiem" at the Queen's Hall for the evening
of January 24, at 8 o'clock, in aid of the Prince Francis
of Teck Memorial Fund.
The Birmingham King Edward VII. Memorial Fund has
now reached ?30,000. The money is to be spent in pro-
viding a statue of his late Majesty, the commission for
which has already been placed, and in building a new
Children's Hospital.
Alderman Sir John Bell will preside at the festival
dinner in aid of the Infant Orphan Asylum at the Hotel
Metropole on Thursday, January 26. A member of the
Stock Exchange has promised a donation of ?1,000 if a
further ?9,000 is collected.
The Committee for the removal of King's College Hos-
pital to South London have received from the proprietors
of the Daily Telegraph a cheque for ?500 and a donation
for a similar amount from the executors of the late Miss
Brandreth in aid of the fund.
The Italian Ambassador, accompanied by the Marchesa
Imperiali, will open the recently built extension of the
Italian Hospital on Saturday, January 7. The new build-
ing, with the site, is the gift of Mrs. Angiola Ortelli,
widow of the founder of the hospital.
Sir Archibald Williamson, in presiding at the annual
meeting of the trustees of Gray's Hospital, Elgin, Aber-
deen, said there was a prospect of a Hospital Sunday
being established in the county. Probation nurses are now
to be engaged for three years, and to be granted certificates
on leaving.
The annual meeting of the Dorcas Society, which is in
connection with the Royal Infirmary, Glasgow, revealed
a deficit of ?83. The Society does Valuable work in
helping convalescents at its store before they leave the
infirmary. In the last twelve months 1,204 convalescents
have been helped in this way.
The Committee of the Birmingham King Edward VII.
Memorial Fund have decided in favour of a site for the
new children's hospital in Ladywood Road. Of the total
amount subscribed ?20,763, or two-thirds, has been
assigned by the donors for the hospital, and ?467 for a
statue of King Edward, which is to be erected in Victoria
Square. The sculptor, Mr. Albert Toft, is to be paid
?2,600 by three instalments.
Recently the Lord Lieutenant of Berkshire (Mr. J. H.
Benyon) presided at a meeting held at Reading to consider
the question of a County Memorial to King Edward.
The memorial will take the form of a children's
ward, to be called the King Edward VII. Ward, at the
Royal Berkshire Hospital at Reading. The foundation-
stone of the new buildings now being built at the hospital
will be laid on February 22 by Lord Ampthill.
Miss Edna May's performance of The Belle of New
York, will take place nightly from February 13 to 18 next,
at the Savoy Theatre, in aid of the Prince Francis of Teck
Memorial Fund for the endowment of the Middlesex Hos-
pital. The private boxes are selling at prices ranging
between 25 and 50 guineas each, and nearly all the stalls for
the first and last nights have been sold. Applications for
seats may be made to Mr. A. C, Abrahams, 36 Hertford
Street, W.
December 31, 1910. THE HOSPITAL 425
The After-Care Association for poor persons discharged
recovered from asylums for the insane appeals for contri-
butions of money and clothing. During 1909 348 cases
were brought to the notice of the Council, and funds are
urgently needed to. carry on and extend the work. The
Secretary is Mr. H. Thornhill Roxby, Church House,
Dean's Yard, Westminster, S.W., and the bankers Union
of London and Smiths Bank, Victoria Street, West-
minster, S.W.
A little while ago Madame Mabel Marsden provided
a very enjoyable entertainment for the patients of the
Cancer Hospital, Fulham Road. Among those who con-
tributed to the programme were Madame Mabel Marsden
herself, whose well-trained contralto voice was heard
to great advantage in "I Think" and "Till Dawn";
and her daughters, Miss Frieda Johnson and Miss
Vera Johnson, the former delighting the audience with
her singing and the latter by her dainty dancing. Valuable
assistance was also rendered by Madame Emilie Steed,
Miss Phyllis Bateman, Miss Hilda Glenister, Mr. Fred
Berlyn, Mr. Harry Briden, Mr. Richard Edwyn, Mr.
Frank Wray, and Mr. H. M. Bateman. Madame Marsden
and her friends are to be congratulated on the success of
their entertainment, which was very greatly appreciated.
The Samaritan Society, in connection with the Western
Infirmary, Glasgow, performs a delicate and useful work
which the hospital itself is unable to touch- At its recent
annual meeting, over which Sir George Beatson presided,
ajid at which Dr. Mackintosh, M.V.O., was present, its
objects were summed up by the former as follows : "To
administer relief which did not fall within the province
of a public hospital, and to supply clothing, surgical instru-
ments, and extra nourishment to patients on returning to
their homes. It gave temporary help to families while their
breadwinners were undergoing treatment in the infirmary,
and endeavoured to find employment for the cured, also
boarding delicate children in the country and sending
patients to convalescent and seaside homes." We need
hardly add that this Society is extremely popular in
Glasgow.
The ceremony of laying the foundation-stone of the New
Coonoor Hospital, " The Lawley Hospital," took place
on September 29, being performed by his Excellency the
Governor. After the presentation by the municipality of
a suitable address to his Excellency, a stone with an
appropriate inscription was lowered into position. His
Excellency then made a speech congratulating the town
of Coonoor on the acquisition of such an institution, and
Major Bryson on the success of his efforts. After sketch-
ing what has been done and has yet to be done in the matter
of the alleviation of suffering and the prosecution of medical
research, the speaker concluded by expressing his appre-
ciation of the compliment paid him in associating his name
with that of the hospital, and wishing success to, and the
blessing of God upon, those directing its destinies. The
site of the hospital has cost 8,000 rupees, and the work
of construction has been paid for by local subscriptions
from ruling native chiefs and others interested, and by a
liberal contribution of 40,000 rupees (as well as a loan of
the balance required) by the Government.
TO HOSPITAL SECRETARIES AND OTHERS.
We invite contributions from any of our readers, and
shall be glad to pay, according to length, for items of news
for the institutional section (including appointments, resig-
nations, gifts, etc.) which we may publish. All payments
for manuscripts used are made as early as possible after
the beginning of each quarter.
The Relaxations of Medical Men.
We shall also be glad to pay for accepted contri-
butions, from any member of the profession, on the
subject of the relaxations of practitioners. This
opens up a wide field, as it includes natural history,
photography, sport, indoor recreations, and motor-
ing. Whenever possible, original illustrations and
photographs should be sent with the MS.
Correspondence.
Correspondence on all subjects is invited, but no
communication can be entertained if the name and
address of the correspondent are not given as a
guarantee of good faith, but not necessarily for pub-
lication. All correspondents should write on one
side of the paper only.
BUSINESS NOTICES.
Annual Subscriptions.
The rate of annual subscriptions to The
Hospital, either direct from the Office or through
agents, is: ?
For the United Kingdom  15s. a year.
For the Colonies and Abroad ... 17s. ,,
For the United Kingdom (with
Insurance Policy)  ?1 ,,
inclusive of postage in each case.
Letters relating to the Publishing, Sale and
Advertisement Departments must be addressed to
the Manager (not to the Editor): ?
The Manager,
The Hospital Building,
28 and 29 Southampton Street,
Strand, London, W.C.

				

